# Budget impact analysis of robotic exoskeleton use for locomotor training following spinal cord injury in four SCI Model Systems

**DOI:** 10.1186/s12984-019-0639-0

**Published:** 2020-01-10

**Authors:** Daniel Pinto, Mauricio Garnier, Jason Barbas, Shuo-Hsiu Chang, Susan Charlifue, Edelle Field-Fote, Catherine Furbish, Candy Tefertiller, Chaithanya K. Mummidisetty, Heather Taylor, Arun Jayaraman, Allen W. Heinemann

**Affiliations:** 1 Department of Physical Therapy, College of Health Sciences, Marquette University, Milwaukee, USA; 2 Department of Medical and Social Sciences, Feinberg School of Medicine, Northwestern University, Evanston, USA; 3 College of Nursing, Marquette University, Milwaukee, USA; 4 Shirley Ryan Ability Lab, Chicago, USA; 5 Department of Physical Therapy and Human Movement Sciences, Feinberg School of Medicine, Northwestern University, Evanston, USA; 6 Department of Physical Medicine and Rehabilitation McGovern Medical School, University of Texas Health Science Center at Houston, Houston, USA; 7 SCI Research, Craig Hospital, Englewood, USA; 8 Department of Physical Medicine and Rehabilitation, University of Colorado, Denver, USA; 9 Spinal Cord Injury Research at the Shepherd Center, Atlanta, Georgia; 10 Division of Physical Therapy, Department of Rehabilitation Medicine, Emory University School of Medicine, Atlanta, Georgia; 11 Craig Hospital, Englewood, USA; 12 Max Nader Center for Rehabilitation Technologies & Outcomes Research, Chicago, USA; 13 Office of Translational Research, Shirley Ryan Ability Lab, Chicago, USA; 14 Spinal Cord Injury and Disability Research, TIRR Memorial Herman, Houston, USA; 15 Pediatrics and Physical Medicine and Rehabilitation McGovern Medical School, University of Texas Health Science Center, Houston, USA; 16 Northwestern University, Evanston, USA; 17 Center for Rehabilitation Outcomes Research, Department of PM&R, Feinberg School of Medicine, Northwestern University, Evanston, USA

**Keywords:** Economic, Budget impact analysis, Spinal cord injury, Robotics, Locomotor training

## Abstract

**Background:**

We know little about the budget impact of integrating robotic exoskeleton over-ground training into therapy services for locomotor training. The purpose of this study was to estimate the budget impact of adding robotic exoskeleton over-ground training to existing locomotor training strategies in the rehabilitation of people with spinal cord injury.

**Methods:**

A Budget Impact Analysis (BIA) was conducted using data provided by four Spinal Cord Injury (SCI) Model Systems rehabilitation hospitals. Hospitals provided estimates of therapy utilization and costs about people with spinal cord injury who participated in locomotor training in the calendar year 2017. Interventions were standard of care walking training including body-weight supported treadmill training, overground training, stationary robotic systems (i.e., treadmill-based robotic gait orthoses), and overground robotic exoskeleton training. The main outcome measures included device costs, training costs for personnel to use the device, human capital costs of locomotor training, device demand, and the number of training sessions per person with SCI.

**Results:**

Robotic exoskeletons for over-ground training decreased hospital costs associated with delivering locomotor training in the base case analysis. This analysis assumed no difference in intervention effectiveness across locomotor training strategies. Providing robotic exoskeleton overground training for 10% of locomotor training sessions over the course of the year (range 226–397 sessions) results in decreased annual locomotor training costs (i.e., net savings) between $1114 to $4784 per annum. The base case shows small savings that are sensitive to parameters of the BIA model which were tested in one-way sensitivity analyses, scenarios analyses, and probability sensitivity analyses. The base case scenario was more sensitive to clinical utilization parameters (e.g., how often devices sit idle and the substitution of high cost training) than device-specific parameters (e.g., robotic exoskeleton device cost or device life). Probabilistic sensitivity analysis simultaneously considered human capital cost, device cost, and locomotor device substitution. With probabilistic sensitivity analysis, the introduction of a robotic exoskeleton only remained cost saving for one facility.

**Conclusions:**

Providing robotic exoskeleton for over-ground training was associated with lower costs for the locomotor training of people with SCI in the base case analyses. The analysis was sensitive to parameter assumptions.

## Background

Between 249,000 to 363,000 persons live with disabilities due to spinal cord injuries (SCI) in the United States, and approximately 17,730 individuals experience new SCI each year [1]. The average age of SCI onset is 43 years, affording the opportunity to resume active community involvement and employment. Despite strong incentives to resume activities, much depends on the restoration of upper and lower limb function. The inability to stand and walk not only limits community involvement and employment but these functional limitations also impose significant secondary health conditions. These conditions include depression, pressure ulcers, severe spasticity, pain, limited joint range of motion, contractures, muscle atrophy, bone loss, and impaired digestive, respiratory, renal, and cardiovascular function [2]. Each condition markedly reduces health and quality of life of persons with SCI. In addition to the physical burden, spinal cord injury accounts for significant health care costs ranging from $368,562 to $1,129,302 in first year costs and $44,766 to $196,107 in annual health care costs depending on the severity of injury [1].

For most individuals, some form of functional limitation persists; however, improvement in mobility and function is possible following the injury. The preservation of volitional motor or sensory function below the lesion level provides an opportunity for motor recovery from intensive training during rehabilitation. This population has great potential for motor recovery and walking. Locomotor training is a standard of care for the rehabilitation of the individual with incomplete SCI. Likewise, walking is usually one of the primary goals of rehabilitation due to physiological and social benefits [3, 4].

Conventional locomotor training strategies include those in which body-weight is supported while clinicians may manually facilitate stepping motions. This training requires multiple personnel to assist with support or lower extremity movement. Although body-weight supported treadmill training (BWSTT) and overground training (OGT) remain common locomotor training strategies, alternative strategies using robotic therapy (RT) devices require less human capital and allow greater repetition than typically is delivered in personnel-intensive approaches [5]. RT may enhance therapists’ productivity and effectiveness while facilitating neurological recovery [5]. RT devices have several advantages, including a structure that provides stability, programmable force-production that facilitates lower extremity movement, [6, 7] precise measurement of forces, [5, 7, 8] and the ability to perform repetitive, labor-intensive tasks [9, 10]. A limitation of many RT devices is their limited interaction with the external environment. Many are stationary and only serve as a tool for the promotion of neural recovery or exercise.

Robotic exoskeletons (RT-exo) are prescription devices comprising an external, motorized orthosis that is placed over paralyzed or weakened limbs for the purpose of facilitating standing, walking, climbing stairs, and performing activities of daily living [11, 12]. Exoskeletons can be used at home, at work, and during rehabilitation [13]. Thus, RT-exo may serve the therapeutic role proposed by stationary robotic devices [14] while allowing mobility and more active community involvement.

### Clinical efficacy

Multiple randomized trials have found outcomes associated with RT to be no different from conventional therapy when similar doses are compared [15–20];; however, a recent meta-analysis of studies investigating RT in individuals with SCI found greater improvement for walking independence (3.73; 95% confidence interval [CI], − 4.92 to − 2.53; *P* < .00001) and endurance (53.32 m; 95% CI, − 73.15 to − 33.48; P < .00001) in robot-assisted training groups [21]. Additional systematic reviews found improvement in locomotor recovery using robot-assisted therapy following SCI [22]. Tefertiller and colleagues reported that RT is likely to be at least as effective as therapy alternatives for locomotor training for individuals with neurological injuries and diseases [14, 22]. Whereas RT-exo has less evidence supporting its use versus treadmill-based RT, several pilot studies of RT-exo have recently been published with preliminary evidence of efficacy [10, 23]. Sale et al. used a prospective, quasi-experimental study, pre-post design and found that RT-exo training resulted in significant improvements in timed up-and-go (TUG), six minute walk test (6MWT), and ten meter walk test (10MWT) [23]. Chang et al. randomized patients to RT-exo versus conventional physical therapy and found the RT-exo group to increase significantly in 6MWT, stride length, and step length after the intervention [10].

RT-exo shares many of the features of treadmill-based RT devices while allowing greater user autonomy and fewer environmental restrictions than treadmill-based RT devices [13]. A recent qualitative study of gym-based RT-exo users identified themes supporting improved community integration [24]; however in a recent narrative overview of RT-exo, the authors critiqued four RT-exo devices, suggesting that they fall short of promises to reengage in activities set in real-world contexts [25]. Furthermore, the authors suggest that RT-exo is better suited for rehabilitation settings than in home settings [25].

### Cost considerations of new technology

New technology in health care is a substantial contributor to rising costs, [26] creating tensions as different stakeholders in the health care sector interpret the value added by the new technology [27]. Health funders attempt to balance cost control, access to treatment, and support for innovation. Health technology producers seek financial reward for their innovations and to fund further development. Patients and clinicians perceive the new technology in terms of benefits produced for the individual [26, 28]. In 1991, Neumann and Weinstein [29] identified ‘five facts’ of new medical technologies that remain true today:

1. On average new technologies improve health outcomes
2. Many new technologies do not improve health outcomes and it is not always easy to discriminate between effective and ineffective technologies.
3. On average new technologies add to health care costs
4. Diffusion of new technology often is inefficient
5. Demand for new technology almost always is high.

The rapid growth and development of robotic devices is an excellent example of such a technology in rehabilitation. The costs of robotic exoskeletons include a high initial purchase price (relative to competing rehabilitation technology), annual maintenance costs, and training costs per physical therapist user. Whereas many new technologies can be cost-inefficient, others have been shown to be cost-neutral or cost-saving [26]. It has been suggested that there is potential for new robotic technology in rehabilitation to be cost saving if the cost of a robotic system could be offset by a decreased need for rehabilitation personnel to conduct the labor-intensive demands of rehabilitation [30]. Whereas robotic exoskeletons initially have been considered a research tool and a device for individual users intent on community use, the robotic exoskeleton is less expensive than stationary robotic devices and may be an attractive option for rehabilitation hospitals as a device for training. An unfortunate reality of technology is the delay between the rising clinical evidence and the rate at which reimbursement changes to account for the costs of technology [31].

### Budget impact analysis – framework

We know little about the budget impact of integrating RT-exo devices into routine therapy services. Carpino et al. conducted a review and model-based cost-effectiveness analysis of lower extremity robotics, showing RT-exo to be cost-effective relative to conventional care and stationary robotics; however, model assumptions have been criticized [32]. Morrison performed an analysis of the financial feasibility of lower extremity robotics, but did not address exoskeletal devices [33]. No budget impact analysis was performed for RT-exo in these economic studies. A budget impact analysis (BIA) provides a framework for synthesizing knowledge when healthcare systems wish to estimate the financial consequences of purchasing new technology [34]. A BIA is not designed to provide a single estimate applicable to all decision-makers, but instead reflects local health systems’ environment and decision-makers’ varying perspectives [34]. For the purpose of this study, we evaluate a model that reflects the introduction or expansion of RT-exo on the total health system budget at four model health systems with different case mixes.

## Methods

### BIA framework and conceptual model for SCI

**Fig. 1:**
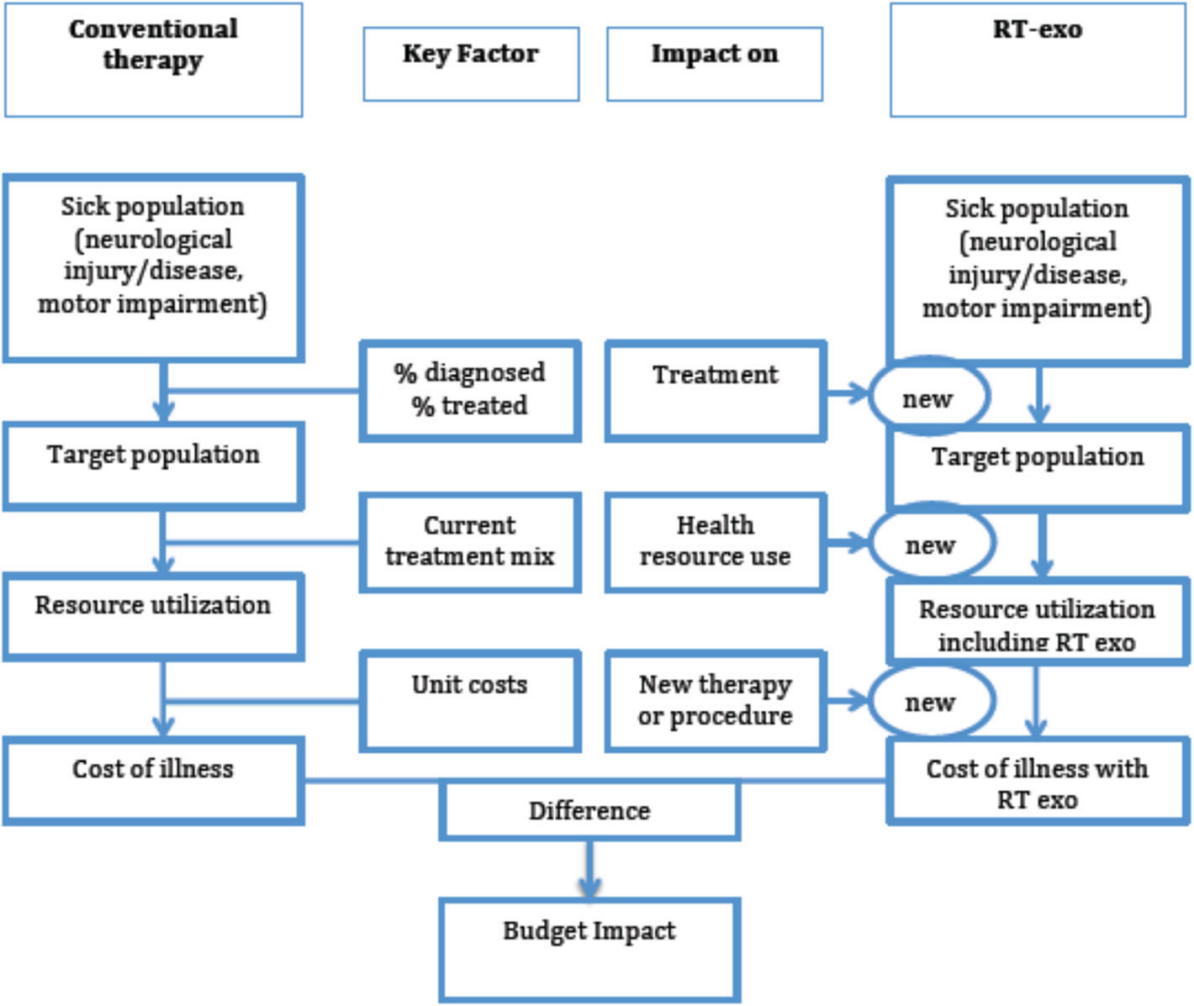
Budget Impact Analysis Schematic

Estimation of the budgetary impact of a new intervention involves a comparison of the “world without” against the “world with” an RT-exo locomotor strategy. The net difference in estimated health-care costs between these two scenarios constitutes the budgetary impact of the intervention. The BIA framework takes into consideration health system features, population eligibility, and resource use in order to understand what current and future costs are for the treatment of a given condition (Fig. 1).

**Fig. 2:**
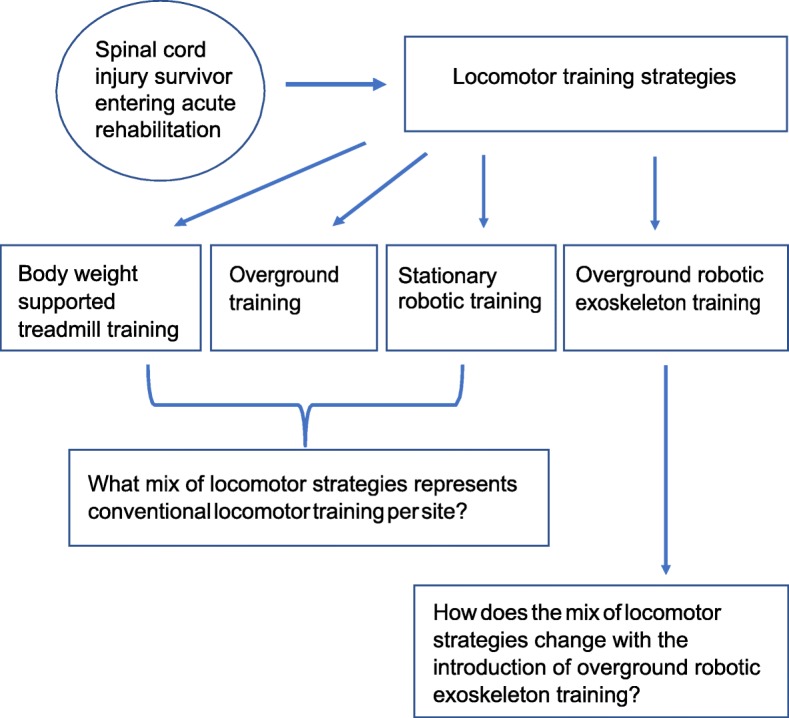
Budget Impact Analysis Conceptual Model for Locomotor Training Market Share

Figure 2 presents the conceptual model for the application of BIA to SCI locomotor strategies, where individuals enter into a rehabilitation facility following SCI and acute medical management. Locomotor training strategies are part of what constitutes rehabilitation following SCI for many patients. Other rehabilitative services include physical medicine and rehabilitation services, occupational therapy, social work, vocational rehabilitation, and psychological services. For the purposes of this analysis, we assume that all other services remain unchanged with the substitution of RT-exo services for other locomotor training strategies.

The perspective of the analysis is from the rehabilitation facilities, specifically Inpatient Rehabilitation Facilities and Long Term Care Hospitals, as the purchasers of technology. A retrospective pre-post study design was implemented to estimate the treatment cost of locomotor strategies. The budget impact of RT-exo was evaluated over one year. The calculation was replicated in a Microsoft Excel-based spreadsheet. Probabilistic sensitivity analysis was conducted using R Statistical Software [35].

Four SCI Model Systems agreed to collaborate on this project: [1] The Shirley Ryan AbilityLab (formerly the Rehabilitation Institute of Chicago), [2] Craig Hospital, [3] Shepherd Center, and [4] TIRR Memorial Hermann. Participating model system sites served as the primary data source for estimates of resource utilization.

### Model inputs

The base case scenario represents the most likely or preferred set of assumptions, and sensitivity analyses explore deviations for this estimate [36]. Our base case model assumptions are presented in Tables 1-3 and Appendix 1. Table 1 presents facility characteristics, estimated number of individuals with SCI receiving locomotor training during the inclusion time period, and locomotor training strategies considered in this exercise. We estimated cost and utilization data from participating Model System sites, details of unit costs and associated sources are included in Appendix 1. The number of individuals with SCI who were estimated to have received locomotor training was multiplied by the average number of training sessions per facility. The total number of training sessions per year was then multiplied by the proportion of training sessions devoted to available locomotor strategies, yielding the number of locomotor sessions per strategy per year. Salary data were populated from the Bureau of Labor Statistics website (https://www.bls.gov/bls/blswage.htm) according to the model system facility location.

**Table 1:** Facility characteristics

Facility label	Hospital structure	Number of individuals with SCI/year eligible for locomotor training	Number of sessions offered per user	Number of locomotor training sessions per year for individuals with SCI
A	LTCH	172	16	2752
B	IRF	248	16	3968
C	LTCH	94	24	2256
D	IRF	155	20	3105

Note: *LTCH* Long Term Acute Care Hospital, *IRF* Inpatient Rehabilitation Facility

**Table 2:** Locomotor strategy cost components in base case analysis

	BWSTT	Stationary robotic	OGT- low cost	OGT– high cost	RT-Exo
Device cost	$70,000	$350,000	$10,000	$225,000	$150,000
Maintenance contract	$8500/yr × 5 years	$15,000/yr maintenance × 5 years	N/A	$7500/yr × 5 years	$10,000/yr
Lifespan	5 years	5 years	10 years	20 years	5 years
Personnel^*^	1 physical therapist (PT), 1 Exercise specialist^†^, 2 aides	1 PT, 1 aide	1 PT, 1 aide	1 PT	1 PT, 1 aide
Training requirement^†^	1 h	5 h	1 h	1 h	18 h

^*^Facility C specified using 1 PT and 3 exercise specialists

^†^We assumed two personnel trained per site

BWSTT, body-weight supported treadmill training; OGT, overground training; RT-Exo, robotic exoskeleton

**Table 3:** Current and projected proportion of locomotor training in base case analysis

	Current locomotor training offerings	Current Market Share per locomotor strategy/number of locomotor sessions*	Future Market Share per locomotor strategy /number of locomotor sessions
Facility A, 2752 locomotor sessions/year	BWSTT	33% / 908	31% / 881
	Stationary robotic	16% / 440	11% / 303
	Over-ground training	51% / 1404	48% / 1293
	Robotic Exoskeleton		10% / 275
Facility B, 3968 locomotor sessions/year	BWSTT	61% / 2420	56% / 2063
	Stationary robotic	5% / 198	3% / 119
	Over-ground training	34% / 1349	31% / 1309
	Robotic Exoskeleton		10% / 397
Facility C, 2256 locomotor sessions/year	BWSTT	34% / 767	30% / 677
	Stationary robotic	33% / 744	30% / 677
	Over-ground training	33% / 744	30% / 677
	Robotic Exoskeleton		10% / 226
Facility D, 3105 locomotor sessions/year	BWSTT	39% / 1211	36%/1118
	Stationary robotic	10% / 311	7%/217
	Over-ground training	51% / 1584	47%/1459
	Robotic Exoskeleton		10%/311

* Number of locomotor sessions per user and locomotor strategy market share is based on facility averages and utilization rates. BWSTT, body-weight supported treadmill training

Table 2 reports BIA inputs and key assumptions concerning the device costs, training costs, and human capital costs associated with locomotor training in inpatient rehabilitation facilities. Where facilities provided more specific data for the analysis, we adapted the inputs accordingly. The duration of all training sessions was assumed to be one hour, including set-up time. We assumed all devices had an adoption rate of 50% across clinicians (i.e., the robotic device sits idle 50% of the time). The adoption rate represents the rate at which the new technology is accepted and the demand for the locomotor training device. We are using assumptions for training session time and adoption rate because RT-exo currently is not used for treatment across facilities.

Table 3 reports the changes in locomotor training strategies between the current and future market share of locomotor strategies within the respective hospital systems. The Appendix provides greater detail of BIA inputs with costs in 2017 USD.

### Sensitivity analysis

Several parameters are likely to influence the RT-exo BIA, including device cost, the choice of locomotor strategy for substitution, and the efficiency of the use of RT-exo. One way sensitivity analyses will vary the following parameters: cost of robotic exoskeleton (50, 200%), adoption rate of robotic exoskeleton device (100–10%), training strategy substituted (highest cost training substituted, lowest cost training substituted), and exoskeleton device life (8 year device life – 3 year device life). A probabilistic sensitivity analysis was performed where uncertainty across all parameters was considered simultaneously. For the probabilistic sensitivity analysis, we considered the uncertainty around human capital cost. Finally, we also tested the assumption of varying conventional locomotor training strategies by 10% (without the inclusion of RT-exo) to assess the influence of varying conventional locomotor strategies on budget impact as scenario analysis.

## Results

In the base case scenario for all hospital systems, offering RT-exo for locomotor training decreased hospital costs associated with delivering locomotor training (Tables 3 & 4). Providing RT-Exo for 10% of locomotor training sessions over the course of the year results in decreased annual costs associated with locomotor training; these savings ranged from $649 (Facility D) to $4784 (Facility B) per annum. The base case scenario had RT-Exo replacing a combination of all currently used robotic services, treadmill training with body-weight support, and over-ground training. It also assumed that RT-Exo was idle 50% of the time.

**Table 4:** Current and projected market share per locomotor strategy

Facility	Current locomotor training offerings	Current Market Share: costs* per locomotor strategy	Future Market Share: costs per locomotor strategy
A: 2752 locomotor sessions/ year	BWSTT	$127,416	$119,700
	Stationary robotic	$66,056	$45,582
	Over-ground training	$104,171	$98,050
	Robotic Exoskeleton	$0	$31,486
Net difference = $294,818 (future costs) - $297,643 (current costs), Savings $2825			
B: 3968 locomotor sessions/ year	BWSTT	$363,747	$333,941
	Stationary robotic	$31,105	$18,890
	Over-ground training	$107,064	$97,627
	Robotic Exoskeleton	$0	$46,674
Net difference = $497,133 (future costs) - $501,916 (current costs), Savings $4784			
C: 2256 locomotor sessions/year	BWSTT	$124,178	$111,783
	Stationary robotic	$115,357	$103,889
	Over-ground training	$58,762	$52,903
	Robotic Exoskeleton	$0	$27,179
Net difference = $295,754 (future costs) - $298,297 (current costs), Savings $2543			
D: 3105 locomotor sessions/year	BWSTT	$174,440	$161,030
	Stationary robotic	$47,037	$33,089
	Over-ground training	$119,009	$109,683
	Robotic Exoskeleton	$0	$35,587
Net difference = $338,993 (future costs) - $340,107 (current costs), Savings $1114			

*Costs are in 2017 USD. BWSTT, body-weight supported treadmill training

### Sensitivity analysis

**Fig. 3:**
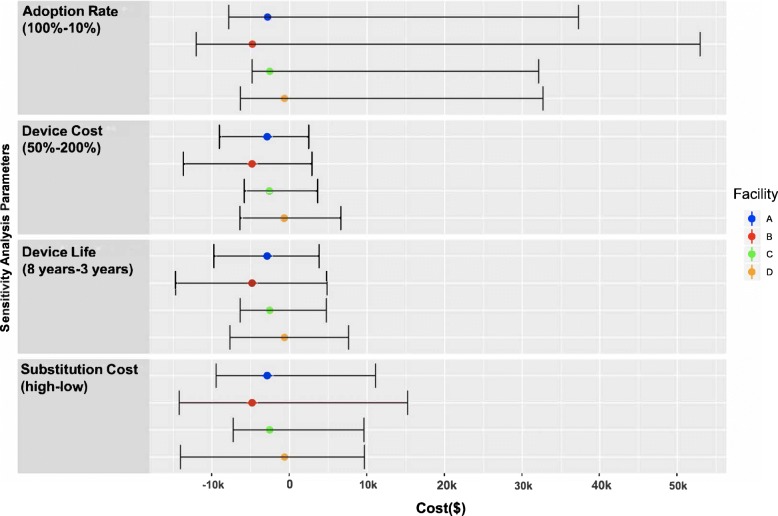
Results from One-Way Sensitivity Analyses. The effect of parameter variation on the base case (depicted as point estimate). Costs are in 2017 USD

The base case scenario was sensitive to several parameters, such as the cost of a robotic exoskeleton, efficiency of robotic exoskeleton use, training strategy substituted, and exoskeleton device life. Fig. 3 plots a series of one-way sensitivity analyses showing the range of costs or savings associated with a change in parameters across each facility. The greatest savings were seen in Facility B ($14,704) where robotic exoskeleton life was extended to 8 years, and the greatest cost was seen in Facility B ($52,934) where robotic exoskeleton adoption rate was limited to 10%. The probabilistic sensitivity analysis simultaneously considered parameter uncertainty of human capital costs (salary), device cost, and locomotor training device substitution. The probabilistic sensitivity analysis produced a range of net budget impacts across facilities from a net savings of $1247per annum in facility C to a net cost of $1620 per annum in facility D. Full probabilistic sensitivity analysis results are presented in Appendix 2.

We explored additional scenario analyses where, instead of choosing to introduce RT-exo, we shift market share to each of the conventional locomotor training strategies by 10%, e.g., in Facility A we assess how locomotor training costs change after we shift from 33 to 43% BWSTT. When stationary robotic and BWSTT are each increased by 10% locomotor training costs increase at all facilities. This is because of the higher personnel cost in BWSTT and higher device cost of stationary robotics relative to the overground training cost. When conventional over-ground training is increased by 10% it lowers costs at all facilities. Full scenario analyses are presented in Appendix 3. For all facilities, the savings from increasing market share to conventional overground training are greater than the savings from shifting market share to RT-exo.

## Discussion

The base case analysis shows small savings that are sensitive to cost or structural components of RT-exo. Despite the expense of RT-exo devices, savings related to a decrease in staffing needs and training for the therapeutic device exist. For hospital systems considering the addition of RT-exo as a locomotor training strategy, those with a higher percentage of human capital intensive strategies such as BWSTT may experience reduced costs if the introduction of RT-exo substitutes for these locomotor training interventions. However, the savings are very small with large degrees of uncertainty.

In the base case scenario, we assumed the robotic overground exoskeleton adoption rate would be 50%. This estimate may be too generous given two factors, the population eligible for RT-exo and clinician acceptance of new technology. RT-exo is a new technology that clinicians could choose not to use for locomotor training despite hospital purchasing decisions. Interestingly, the base case scenario was more sensitive to assumptions on clinical utilization parameters than device-specific parameters. For example, limiting the RT-exo useful life to 3 years (versus 5) only resulted in an increase in locomotor training cost per annum of between $3847 and $7611 across facilities. However, if RT-exo was adopted at a rate of 10%, i.e., the device is idle 90% of the time (as opposed to the 50% used in our model), annual costs of locomotor training would increase between $32,096 and $52,934 across facilities. When considering all sensitivity and scenario analyses the least costly alternative is when over-ground training sessions are increased by 10%. This makes intuitive sense because device costs and personnel costs are lowest. Over-ground training is a well developed and clinically integrated technology, therefore few efficiencies can be expected from changes in technological adoption and further integration into clinical practice. Conversely, further technological development stands to improve the business case for RT-exo but barriers in adoption and integration can impair its efficiency. Issues of technological adoption warrant additional attention.

Turchetti et al. provide a comprehensive overview of technology adoption with application to robotic devices in rehabilitation [31]. The authors discuss barriers organized around technological, behavioral, organizational, and economic factors. Technological factors include ease of use, level of training necessary for caregivers to master the technology, and the anthropomorphic design of the product [31]. Of particular note with technological barriers is the shift from roles once played by the clinician to the device that may create behavioral barriers to adoption. Learning new technologies requires effort on the part of clinicians, patient and caregivers, and resistance to change is a common phenomenon. Organization factors may be manifested in the need for redistribution of responsibilities and establishing a new organizational balance. Economic barriers include the dearth of cost-effectiveness data and the discordance between the timing required for purchasing decisions and the rate of change concerning reimbursement.

Another barrier to adoption is the different perspectives on the value added by the new technology suggesting the need for further economic studies that consider multiple perspectives in order to address the interests of all stakeholders [31]. Swank and colleagues report on the feasibility of integrating RT-exo as a part of inpatient rehabilitation for an individual with SCI and illuminate on many aspects of RT-exo adoption identified above [37]. Of note was the need for additional training for intra- and inter-physical therapist consistency with use of RT-exo despite being certified in use of the device. Working with hospital administrators and support staff was needed to address workflow issues such as optimizing device sizing and adjust settings prior to the rehabilitation visit.

We did not quantify several key considerations regarding RT-exo in this analysis. Hospital systems may invest in RT-exo as a means of building or maintaining their reputation as a leader in the use of cutting-edge technology. In this model, RT-exo is considered only as a substitute for locomotor training in the rehabilitation setting; however, there is potential for RT-exo to augment some community mobility. Devices such as RT-exo are likely to decrease in cost with greater competition in the market [38]. Numerous robotic device companies are entering the market with several at substantially lower price points than the price weight used for this investigation, e.g., $30,000 instead of $150,000 [39]. One device manufacturer anticipates being able to produce RT-exos at $10,000 – $15,000, [39] which would be comparable with the price weight used for our low cost OGT device. With the use of new medical devices, there is a device-operator interaction known as the ‘learning curve” effect [40, 41] during which inefficiencies are more likely; this is arguably the state that we are currently in with respect to RT-exo use in rehabilitation and we anticipate an improved business case in the future.

### Study limitations

This project did not take into account differences in locomotor training effectiveness between alternatives. There is no strong evidence showing the superiority or non-inferiority of RT-exo. The evidence for the comparative effectiveness of robotic overground exoskeleton for locomotor training in SCI is limited, but several pilot studies show promising results [10, 23], and multiple clinical studies are underway or in development that will add to the evidence base (ClinicalTrials.gov Identifier: NCT03340792, NCT03477123, NCT02322125, NCT03443700). Therefore, the findings of this BIA must be interpreted with caution because we assume non-inferiority. In addition, there is limited data on the risks to users with SCI and personnel associated with locomotor training for RT-exo relative to other locomotor training strategies [12]. The retrospective study design provides estimates for the number of individuals with SCI who received locomotor training therapies in 2017. Individuals with high cervical incomplete injuries who do not have upper extremity strength are not always eligible to use all RT-exo systems, but they are eligible to receive locomotor training with one RT-exo device and with other interventions. Therefore the volume of eligible patients may be lower than our estimates suggest; however, devices under development are designed to be used with individuals who have higher levels of SCI [42]. We may have overestimated eligibility across therapy types due to varying eligibility criteria specific to each device (e.g., robotic exoskeleton device fit versus BWSTT fit criteria).

## Conclusions

The assessment of economic efficiency-with robotic exoskeleton is in its infancy. Our model provides a framework upon which economic studies can build. Utilizing RT-exo for locomotor training may be an efficient use of hospital resources if key assumptions concerning efficacy hold true and the technology is adopted within the health system; however, a large degree of uncertainty surrounds our estimates as evidence for differences in the effectiveness of among approaches is limited.

## Data Availability

The dataset used and analyzed during the current study are available from the corresponding author upon request.

## Appendix 1

**Table 5:** Unit costs and associated sources

Component	Cost/unit	Assumptions and sources
Locomotor strategies		
RT-exo	$150,000	5 year life-span+Annual maintenance contract ($10,000) × 5 years
Litegait - overground training	$10,000	Rehabilitaton hospital purchasing department
Zero G track system	$225,000	Rehabilitaton hospital purchasing department+Annual maintenance contract ($7500) × 5 years
Treadmill training with body-weight support (BWS) - treadmill and harness system	$70,000	Rehabilitaton hospital purchasing department +Annual maintenance contract ($8500) × 5 years
Lokomat	$350,000	$350,000 + Annual maintenance contract ($15,000) × 5 years
		Rehabilitaton hospital purchasing department
Personnel		
PT	$53.95 – Facility A	2017 BLS mean hourly rate by state + 30% benefits https://www.bls.gov/ooh/healthcare/physical-therapists.htm
	$56.68 – Facility B	$41.50*0.3 – Facility A
	$58.08 – Facility C	$43.60 + 0.3 - Facility B
	$51.58 – Facility D	$44.68 + 0.3 - Facility C
		$39.69 + 0.3 – Facility D
PT Assistant	$33.66 – Facility A	2017 BLS mean hourly rate by state + 30% benefits
	$36.30 – Facility B	25.89 + 30% benefits – Facility A
	$43.68 – Facility C	27.92 + 30% benefits – Facility B
	$34.61 – Facility D	33.60 + 30% benefits – Facility C
		26.62 + 30% benefits – Facility D
Exercise specialist	$31.94 – Facility A	Exercise physiologist
	$34.46 – Facility B	2017 BLS mean hourly rate by state + 30% benefits
	$28.90 – Facility C	$24.57 - Facility A
	$31.87 – Facility D	$26.51- Facility B
		$22.23 - Facility C
		$23.99 - Facility D
PT Aide/tech	$17.04 – Facility A	2017 BLS mean hourly rate by state + 30% benefits
	$19.44 – Facility B	$13.11+ 30% benefits – Facility A
	$16.76 – Facility C	$14.95+ 30% benefits – Facility B
	$20.15 – Facility D	$12.89+ 30% benefits – Facility C
		$15.50 + 30% benefits – Facility D

## Appendix 2

**Table 6:** Full results of Probabilistic sensitivity analysis

	Mean (SD)	95% CI
Facility A		
Current cost	318,727.09 (38,750.52)	244,101.81, 396,054.92
Future cost	319,901.66 (37,082.70)	248,223.86, 392,349.78
Difference	1174.56 (13,552.87)	−23,305.15, 30,106.35
Facility B		
Current cost	340,331.06 (39,527.96)	264,223.77, 418,017.98
Future cost	340,533.79 (37,800.31)	266,498.82, 414,476.83
Difference	202.73 (13,430.07)	−24,508.92, 28,976.15
Facility C		
Current cost	355,740.28 (39,565.62)	279,999.34, 432,895.62
Future cost	354,493.63 (37,927.97)	280,420.54, 428,026.15
Difference	− 1246.65 (13,663.67)	− 26,749.97, 27,183.83
Facility D		
Current cost	318,888.07 (39,101.88)	242,958.61, 395,990.05
Future cost	320,508.13 (37,354.01)	247,590.25, 393,909.77
Difference	1620.06 (13,422.23)	− 7404.26, 31,122.70

*CI* Credible Interval

## Appendix 3

**Table 7:** Scenario Analysis where conventional training strategies (pre RT-exo) are fluctuated by 10%

	Current locomotor training offerings	Current Market Share per locomotor strategy/number of locomotor sessions*	Future Market Share per locomotor strategy /number of locomotor sessions - 10% increase in body weight support	Future Market Share per locomotor strategy /number of locomotor sessions - 10% increase in Stationary robotic	Future Market Share per locomotor strategy /number of locomotor sessions - 10% increase in Overground training
**Facility A,** 2752 locomotor sessions/year	Body-weight support	33% / 908	43% / 1183	28% / 771	28% / 771
	Stationary robotic	16% / 440	11% / 303	26% / 716	11% / 303
	Over-ground training	51% / 1404	46% / 1266	46% / 1266	61% / 1679
**Facility B,** 3968 locomotor sessions/year	Body-weight support	61% / 2420	71% / 2817	56% / 2222	55% / 2182
	Stationary robotic	5% / 198	1% / 40	15% / 595	1% / 40
	Over-ground training	34% / 1349	28% / 1111	29% / 1151	44% / 1746
**Facility C,** 2256 locomotor sessions/year	Body-weight support	34% / 767	43% / 970	29% / 654	29% / 654
	Stationary robotic	33% / 744	29% / 654	43% / 970	28% / 632
	Over-ground training	33% / 744	28% / 632	28% / 632	43% / 970
**Facility D**, 3105 locomotor sessions/year	Body-weight support	39% / 1211	49% / 1521	36% / 1118	34% / 1056
	Stationary robotic	10% / 311	5% / 155	17% / 528	5% / 155
	Over-ground training	51% / 1584	46% / 1428	47% / 1459	61% / 1894
**Facility**	**Current locomotor training offerings**	**Current Market Share: costs* per locomotor strategy**	**Future Market Share: Body weight support**	**Future Market Share: Stationary robotics**	**Future Market Share: Overground training**
**A:** 2752 locomotor sessions/ year	BWS	$127,416	$165,994	$108,127	$108,125
	Stationary robotic	$66,056	$45,582	$107,004	$45,581
	Over-ground training	$104,171	$93,969	$93,969	$124,568
	subtotal	$297,643	$305,545	$309,099	$278,274
		**Net difference**	**$7902**	**$11,456**	**$–19,359**
**B:** 3968 locomotor sessions/ year	BWS	$363,747	$423,360	$333,941	$327,980
	Stationary robotic	$31,105	$6674	$92,182	$6674
	Over-ground training	$107,064	$88,190	$91,336	$138,520
	Subtotal	$501,916	$518,224	$517,459	$473,175
		**Net difference**	**$16,308**	**$15,542**	**$–28,742**
**C:** 2256 locomotor sessions/year	BWS	$124,178	$160,172	$108,061	$108,061
	Stationary robotic	$115,357	$100,446	$148,656	$97,002
	Over-ground training	$58,762	$49,384	$49,384	$75,777
	subtotal	$298,297	$310,001	$306,101	$280,840
		**Net difference**	**$11,705**	**$7804**	**$–17,457**
**D:** 3105 locomotor sessions/year	BWS	$168,627	$211,837	$155,665	$147,023
	Stationary robotic	$47,037	$23,791	$79,581	$23,791
	Over-ground training	$119,009	$107,352	$109,683	$142,323
	subtotal	$334,673	$342,980	$334,929	$142,323
		**Net difference**	**$8307**	**$10,256**	**$–21,537**

* negative (−) values denote a net saving

## Abbreviations

BIA: Budget Impact Analysis
BWSTT: Body-weight supported treadmill training
IRF: Inpatient Rehabilitation Facility
LTCH: Long Term Acute Care Hospital
OGT: Overground training
PT: Physical therapist
RT: Robotic therapy
RT-exo: Robotic exoskeleton
SCI: Spinal Cord Injury
